# Parental appearance teasing in adolescence and associations with eating problems: a systematic review

**DOI:** 10.1186/s12889-021-10416-5

**Published:** 2021-03-06

**Authors:** Lucy M. Dahill, Stephen Touyz, Natalie M. V. Morrison, Phillipa Hay

**Affiliations:** 1 Translational Health Research Institute, Western Sydney University, Locked Bag 1797, Penrith, NSW 2751 Australia; 2 Department of Psychology, University of Sydney, Sydney, Australia; 3 School of Medicine, Western Sydney University, Penrith, Australia

**Keywords:** Adolescents, Teasing, Body image, Eating problems, Eating disorder, Parent child communication, Appearance teasing, Weight teasing, Shape teasing, Family processes

## Abstract

**Background:**

The adolescent years see significant physical and emotional development that lay foundations for patterns of behaviour that can continue into adult life, including the shaping of eating behaviours. Given parents are key socio-environmental drivers and influencers of adolescent behaviours around physical health and wellbeing, it is critical to consider if specific forms of parental communication are potentially contributing to the associated emotional difficulties experienced in the adolescent years. The aim of this research was to systematically review the myriad of literature pertaining to the prevalence of parental weight or appearance-based teasing and adolescent eating problems to examine how the scientific and clinical community currently understands the relationship between these domains.

**Methods:**

A systematic search of the literature, using the SCOPUS, APA PsycINFO, Medline, CINAHL databases, reference lists and Google Scholar, was undertaken to identify relevant literature for parental teasing and problem eating in adolescents aged 10–19 years, published between January 1980 to October 2020, in English or French.

**Results:**

Six studies met criteria for inclusion, all were cross-sectional studies and two included additional prospective data. Although parents were not the most common perpetrators of teasing, often subsidiary to that of peers and siblings, the influence and impact of parental teasing remained significant, and in some cases, appeared to interact with sibling-based teasing. This teasing was associated with problem eating behaviours for adolescents.

**Conclusions:**

There is evidence in the literature to suggest the existence of an association between ‘eating problems’ amongst adolescents and exposure to parental appearance or weight teasing. Parents are unlikely to be aware of the perception or impact of the words they use or the wider influence these words may have. Future research should employ representative longitudinal designs to develop a greater understanding of the relationships between parental communications around their adolescent’s appearance or weight and how that communication is perceived by adolescents within complex family processes.

**Trial registration:**

PROSPERO 2018 CRD42018109623. Prospectively registered 15th October 2018.

**Supplementary Information:**

The online version contains supplementary material available at 10.1186/s12889-021-10416-5.

## Background

According to the World Health Organisation (WHO), adolescence, defined between 10 and 19 years of age, [1] is a time of rapid physical, emotional and cognitive development which creates a unique population requiring age specific attention to development and health [2]. It is during this period of development that foundations are set for patterns of behaviour that can continue into adult life [2], of note, the shaping of eating behaviours [3–5]. Parents and primary caregivers are key socio-environmental drivers and influencers of childhood and adolescent behaviours, anxiety, self-esteem and self-efficacy around health and wellbeing [6–10]. The value of a parents’ opinion, as a relationship of significance to adolescents, influences the quality of family relationships as well as their position as role models in their adolescent’s life. However, Randal Day writes on family processes, ‘the interaction in families is larger than any one person or even any one rule or pattern’ [11, 12]. Therefore, although it is not possible to say that parents are the cause of an adolescent’s eating problems, when the person who is their primary carer is criticising through body shaming, or worse, perceived to be rejecting them through likely misunderstandings in communication styles such as humour, teasing and jokes, the potential for adverse impact is worth considering as a possible influence or association [13–15]. Parental rejection is found to be positively associated with concurrent emotional eating [16] and related to psychological distress and anxiety in later adult life [7, 10, 17]. Such repeated emotional and behavioural experiences in adolescence may contribute to ‘eating problems’ in adulthood creating health, logistical, and fiscal burdens on the health system, the community, the individual and their family system [18]. Critically, this pathway has long-term sequelae including a substantially elevated risk of anxiety and depressive disorders, cardiovascular disease, chronic fatigue and chronic pain [10, 17, 19, 20].

The growing evidence lends itself to a simple stepped illustration: if you are underweight or overweight (clinically or not) in adolescence you are more likely to be teased by a mix of sources [20, 21], with such teasing being positively related to anxiety [10, 17, 22, 23], and such anxiety directly affecting self-esteem [10, 23] and indirectly affecting eating through the use of ‘unhealthy’ or ‘problem’ eating behaviours as a coping mechanism. Therefore, this review will consider the prevalence of parental teasing, what parents teased about, the influence of parental teasing and any associations with eating disorders as an outcome. Even though the outcome of interest in this review is eating disorders, we will make note of psychological and psychosocial associations as significant predictors for unhealthy eating more generally in adolescence.

### Eating disorders, disordered eating and “eating problems”

An *Eating Disorder* is a clinical categorisation that represents a grouping of eating-based behaviours that are deemed to differ from societal norms, including: anorexia nervosa, bulimia nervosa, binge eating disorder and other specified and unspecified eating disorders; these involve obesity, disturbed body image and / or weight loss behaviours [24]. The term *disordered eating* is typically used to allow a more expansive consideration of eating based problems that are broader than clinical diagnoses – for instance, “emotional eating” where overconsumption of food becomes a method of mood regulation [25] but which, in itself, is not cause for an eating disorder diagnosis. For this review, the term ‘*eating problems*’ is used as our outcome to encapsulate the diversity of terms used in the literature on parental teasing on weight, shape and or body and to cover both clinical and non-clinical descriptions of problematic eating behaviours in diverse samples of adolescents [9, 26–28].

### Family processes and communication

Family processes consider the family as a system, a collection of interacting parts that are trying to attain a common goal and to achieve those communal aims, families are likely to repeat certain routines or patterns of behaviour; family processes consider all family members need to be fully subscribed to the goal for it to be achieved [11]. Previous research has considered family processes as a moderator for dietary behaviours [29] and influence for these family goals has previously been considered in light of the Tripartite Influence model [30]. Characteristics of family processes (such as family functioning, cohesion and conflicts), can contribute to an interplay between other characteristics (such as age, gender) and intrafamilial processes (such as parent-parent and parent-child relationships), and can influence associations and outcomes [29]. However, there have been dramatic shifts in the structure of families over the past few decades with increases in prevalence of single-parent and blended families, and the family structure is now extremely varied [31]. Family processes might also influence the way communication is heard in families. Krauss and Fussel, 1996 succinctly offer ‘Communication is more than just talking, it is the process by which meaning is created and managed’ [12]. Within families, this communication is complex (as is the family structure) and may be overt (obvious, explicit, observable and visible) or covert (more concealed, not obvious, more subtle and harder to read) [11, 32]. Adolescents, like parents, can sometimes decode messages incorrectly and messages may neither be sent nor received in the way they were intended [11].

### Teasing

We specifically wanted to review the literature using ‘teasing’ as an intervention because it is a complex form of communication whose definition can vary between situations being used by a parent (our proposed context). Teasing is described as a specific type of bullying characterised by verbal taunts as a form of provocation (criticism or hostility) that is mitigated by off-record markers (playful gestures) - both of which may be present by varying degrees [33–35]. It requires the ability to understand intention, social context, non-verbal and non-literal communication [33, 34]. The level of emotional maturity required to process and contextualise this language is found in later childhood development and as such, the “humour” of teasing, as used by adults, may be comprehended by children in a far more literal and concrete manner. Furthermore, the impact of teasing can be cumulative, and teasing from multiple sources such as siblings and peers, or across a long period of time, puts children and subsequently, their adolescent self, at an even higher risk of more submissive behaviour, more unfavourable social comparisons and more emotional health problems [36–38] including eating problems [39, 40]. Considered in the context of peer victimization, Hayden-Wade et al., [41] found teasing to be the most psychologically harmful of most communicative forms of bullying and harassment. Vandewalle et al., found that it was parental rejection, over peer rejection, that was found to be uniquely and positively associated with emotional eating [16] therefore, teasing specifically from parents is likely to be similarly detrimental and impactful and contribute to, not cause, the equifinality of eating problems.

Whilst a previous systematic review and meta-analysis of bullying and teasing associations with eating disorders has found those with eating disorders were significantly more likely to have been teased prior to the onset of their eating disorder, there were very few that considered the source of the teasing [42]. Therefore, to further our understanding of potential prevention strategies for families dealing with eating disorders and their onset in adolescents, it is valuable to consider ‘teasing’ as a particular form of communication from parents. Parental teasing in this review will focus on appearance or weight based/related teasing, hereafter referred to as A-RT and W-RT, unless a particular paper only studies weight-based teasing (W-BT), as well as any influence parental teasing has on other members of the household.

In this review we study adolescence defined by the World Health Organisation as between 10 and 19 years of age [1]. It is of particular interest as a stage of development synonymous with a higher prevalence of parent-child conflict [43] and it is a key period for establishing positive relationships with, and communications regarding, emotions and eating [4].

### Objective

Whilst the impact of parental teasing on children under 10 years of age has been examined [44] there has not, to our knowledge, been a systematic synthesis of empirical findings to inform understanding of how parental teasing during, or continuing through, adolescence impacts on eating problems. Thus, this systematic review aimed to investigate the association between exposure to parental appearance related teasing (A-RT) and or weight related teasing (W-RT) and eating problems amongst adolescents.

## Method

The review is reported using the PRISMA guidelines, [41]. A protocol was developed and established prior to conduct of the review registering a review question, a search strategy, inclusion and exclusion criteria, a risk of bias assessment, a plan for data extraction and a strategy for data synthesis. This strategy was prospectively registered on PROSPERO International Prospective Register of Systematic Reviews PROSPERO 2018 CRD42018109623 (https://www.crd.york.ac.uk/PROSPERO/display_record.php? RecordID = 109,623). Minor changes to the protocol and refining of the review question were made following the preliminary searches. This was because the preliminary searches generated a very high number of irrelevant results.

### Eligibility criteria and study selection

Based on an understanding of previous research in the field of parental communication with adolescents and disordered eating, studies were included in this review if they included (i) a participant population of adolescents who were exposed to appearance or weight related teasing from parents, these two specific forms of teasing have validated measures such as the Perceptions of teasing scale (Thompson 1995) and Weight Based Victimization Questionnaire (Puhl et al., 2011) and were considered to offer a standardized reporting of parental teasing in relation to influences for disordered eating (ii) where the exposure to appearance or weight related teasing occurred during adolescence (aged between 10 and 19 years of age, the age the World Health Organisation define as adolescence). If a paper included participants outside the age range, for example 9 year or 20 years but these participants made up less than 10% of the sample and the mean age was within the selected age range these studies were to be included. If this was not clear the authors were to be contacted for confirmation and (iii) where the eating problems were identified as an outcome, via a validated instrument, or other assessment of weight, shape or body overvaluation or other body image cognitions such as the Eating Disorder Examination Questionnaire (Fairburn et al. 1994) or the Eating Disorder Inventory (Garner 1991). We inspected studies that included an observation of known psychopathology of eating disorders. However, if they did not specifically report eating disorders with a validated measure, those studies were not included in the final review. To increase sensitivity and capture as many studies as possible, both qualitative, quantitative, case studies and original full research studies were included in the original search. Although we did not set out to preclude Randomized Control Trials (RCTs), no RCT’s were appropriate for inclusion. See Table 1.

**Table 1:** Inclusion and Exclusion Criteria

Selection Criteria	Inclusion Criteria	Exclusion Criteria
Population	Adolescents as defined by the World Health Organisation (WHO), between 10 and 19 years of age. Include teenagers, teens or youth.	Non-related adolescent/teen/youth articles. If the weight, shape, eating teasing happens outside adolescence - younger than 10 or older than 19.
Intervention	Teasing specific to appearance / weight / body image. Appearance based teasing. Weight based teasing. Include verbal bullying.	Article not specifically reporting teasing or verbal bullying as a specific construct. Not reporting appearance or weight-based teasing.
Context	Parents. Include parental / mother / father.	Article does not report specifically on parents. If teasing was from peers, teachers, healthcare workers, siblings.
Outcome	Disordered eating as an outcome or association. Must include a validated measure of disordered eating. Includes eating disorder psychopathology	Article does not report disordered eating as an outcome or association Article does not include a standardized measure of disordered eating.
Study type	Qualitative or Quantitative including case study designs, cross-sectional, longitudinal and randomized control trials. Original Full Research Studies.	Non-original studies, book reviews, opinion pieces, non-peer reviewed journals, unpublished theses.
Language	English and French Language Studies	Non-English or French language studies.
Date range	Studies from 1980 to present 1980 is when the Diagnostic and Statistical Manual of Mental Disorders, Third edition (DSM-III, 1952) first included Bulimia Nervosa and signified the modern era of eating disorders.	Being older that 1980.

### Information sources and search

We used the PICO framework to consider adolescents’ (population) appearance teasing (intervention) by parents (context) and associations with eating problems (outcome) [45]. The following electronic databases were searched based on research of appropriate databases for our area of interest: Ovid for Medline, EBSCOhost for CINAHL and APA PsycINFO, SCOPUS. Google returned results based on its own relevance algorithm, we search the first four pages (20 results per page) of Google Scholar for any other related articles. The first search considered a variety of search terms to establish our population (adolescents, teens, teenagers, youth); our intervention (teasing, bullying, verbal abuse, jokes, victimization); what the teasing was about (appearance, weight, shape, body); our context (parents, parental, maternal, paternal, mother, father); and outcome (eating disorder, eating problems, anorexia, bulimia, binge eating). We considered keyword and text searches to increase the likelihood of accurate results with adjustments made based on the protocols of each database. We manually searched reference lists for included studies, systematic reviews and meta-analyses that focused on teasing to increase sensitivity for parental teasing articles. No new articles were found [see Additional file 1 for a specific list of search terms used across each database]. The Boolean operator ‘OR’ was used to explode and map the Medical Subject Headings (MeSH) terms. As an example, the specific search phrase we used for Scopus was ((TITLE-ABS-KEY (adolescen*) OR TITLE-ABS-KEY (teen*) OR TITLE-ABS KEY (youth*)) AND PUBYEAR > 1979) AND ((TITLE-ABS KEY (teasing) AND TITLE-ABS KEY (appearance OR body OR shape)) AND PUBYEAR > 1979) AND ((TITLE-ABS-KEY (parent*) OR TITLE-ABS-KEY (maternal) OR TITLE-ABS- KEY (paternal)) AND PUBYEAR > 1979) AND ((TITLE-ABS-KEY (“eating problems”) OR TITLE-ABS-KEY (anorexia) OR TITLE-ABS-KEY (bulimia) OR TITLE-ABS-KEY (“eating problems”)) AND PUBYEAR > 1979).

**Fig. 1:**
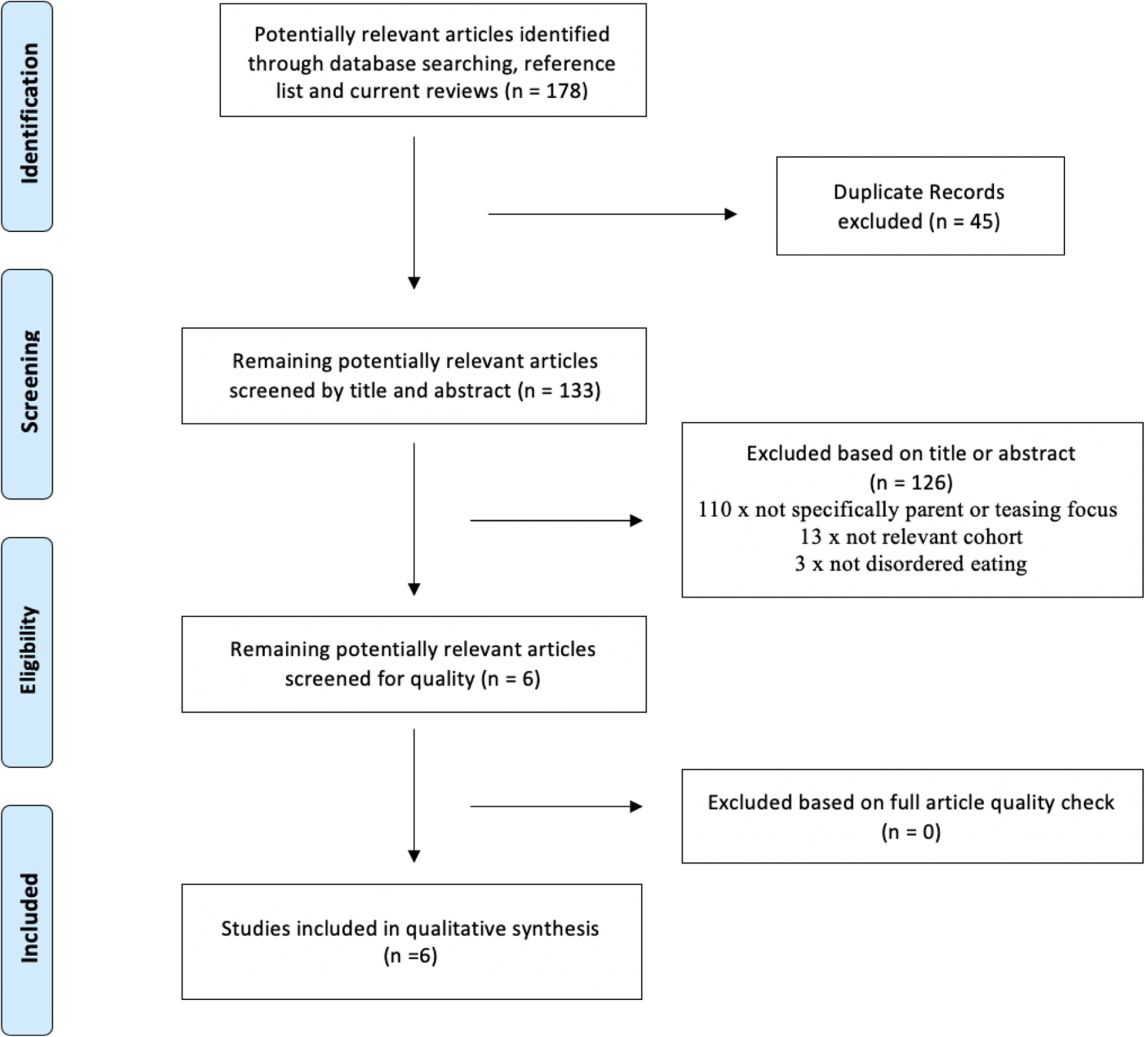
PRISMA flow chart of included and excluded studies

Subsequently these terms were then combined using the Boolean ‘AND’ operator. Finally, a year filter was applied to limit the returned articles to the time-period between 1980 and 2020. 1980 is when the Diagnostic and Statistical Manual of Mental Disorders, Third edition (DSM-III, 1952) first included Bulimia Nervosa and signified the modern era of eating disorders. To accommodate the researchers’ language competencies the search was restricted to English and French language articles [see Table 1]. In an attempt to increase sensitivity, reference lists of included articles, prior systematic reviews and meta-analyses in this domain were manually screened for relevant studies. In the first stage, all article titles and abstracts were screened for relevant keywords, relevance to the specific research question and inclusion and exclusion criteria (LD). In the second stage two authors (LD and PH) performed a full-text review of those remaining articles to confirm they met the eligibility criteria. If there was a disagreement about eligibility, these were to be resolved through discussion with a third reviewer (ST). A detailed list of records found by database [see Additional file 3] is mapped onto the PRISMA flow diagram [see Fig. 1]. We conducted this search within 24 months of completing the review and repeated the search in March and July 2020 to ensure a comprehensive literature search.

### Risk of Bias in individual studies

The quality of the studies to be included in this review was assessed using the 16 items relevant to cross-sectional studies from the Downs and Black Quality Index (DBQI) [46]. Items were scored ‘Yes’ [1] or ‘No/unable to determine’ (0). They included a measure of the studies effectiveness in the following areas: nine questions pertaining to reporting, three questions pertaining to external validity, one question pertaining to internal validity and power each and two other questions related to our inclusion and exclusion criteria namely ‘Was it an original study?’; ‘Did the study use a validated measure of disordered eating?’. The maximum score was 16 with higher scores indicating more robust methodology [see Additional file 2 for full Variation of Downs and Black Checklist]. A sensitivity analysis was planned to test the impact of removing any potentially poor quality studies with a score < 10 but this was not required.

## Results

### Study selection

As shown on Fig. 1, the initial database search yielded 178 records. 45 duplicates were identified and immediately excluded. Through title and abstract review, a further 126 records were then excluded.

Reasons for exclusion were decided in a systematic order, excluded because they were not specific to parent teasing as a construct (i.e. focused on family, sibling or peer teasing) or did not have a teasing focus, (i.e. weight commentary rather than teasing) *n* = 110; excluded for not being from the cohort (i.e the cohort were younger than 10 years or older than 19 years) *n* = 13; excluded for not measuring disordered eating *n* = 3. Results of a hand-search of included paper, existing meta-analyses and systematic reviews resulted in no new articles but duplicates of the final included studies and duplicates of excluded papers. Full text articles were obtained for the remaining six records and a more detailed evaluation based on inclusion and exclusion criteria and quality assessment was undertaken by LD and PH. There were no disagreements and all 6 remaining texts were included in the qualitative synthesis. Of the 178 studies originally identified, six studies met the criteria outlined for this systematic review. [see Additional File 3 for reference list for included articles].

The six studies included in this review displayed a Median of 15 using the DBQI measure with an interquartile range demonstrating the spread of the results between 14 and 15. There was statistical ambiguity around the age of participants in one of the five studies, which suggested the inclusion of nine-year-old children in their cohort, therefore outside the inclusion criteria for this review which was specifically interested in the adolescent population due to the perceived increase in parental conflict at this age. The authors of the concerned paper [27] were contacted for clarification of age spread. The primary author responded and advised the included nine-year-olds represented less than 10% of the cohort. Based on this information, the study was retained in this review. All six included studies had low risk of bias with regards to describing the principle confounders in each group and using appropriate analyses for confounding [See Additional file 2 for full Variation of Downs and Black Checklist].

### Risk of bias across studies

All six research papers included in this review were from peer-reviewed journals and were cross-sectional, with two including additional prospective data. All studies used non-clinical samples with two concentrating on minority populations. Four studies included mixed gendered populations, the balance focused on girls. Each study reported on funding sources and none were considered a conflict of interest or a source that could influence results presented in the studies. No studies used mixed methods and/or qualitative methodology.

### Study characteristics

As shown in Table 2, the studies included in this review captured cohorts from America, Australia and Germany. One study was from a female only general population cohort [9]; two studies were mixed gender general population studies [26, 48]; one study was mixed gender population with binge-eating disorder (BED) and had a comparator group [47] another concentrated specifically on a Hispanic and African American demographic [27] and another study captured adolescent military dependents [28]. Of note, 91% of the adolescents in the review came from the single study [26]. Gender spread across studies was not equal with the total adolescents included in this review (*N* = 11,644) consisting of 59% girls (*n* = 6887). Boys in the review equated to 41% (*n* = 4757), with 95% of these male participants coming from the single study [26] study. The two papers that considered parent gender were both with a female only population [9, 27].

**Table 2:** Characteristics of Included Studies

Studies	Aims	Sample	Methodology
Authors (Year) (Country)	Specific to parent teasing	Number, Population, Mean Age	Study style; Statistical analysis used; What adjusted for; Specific teasing and eating pathology and teasing measures.
Haines et al. (2010) [26] (USA)	To identify shared risk and protective factors for purging, binge eating and overweight.	10,540 mixed gender (n=6022 female) offspring of Nurses’ Health study (NHS II) and Growing Up Today Study (GUTS) 11-17 years of age. Female *M*=13.9 (*SD*=1.6) Male *M*=13.8 (*SD*=1.5)	Cross Sectional Prospective design; Generalized estimating equations; Weight-Related Teasing (W-RT); Self-report questionnaires: Youth risk Behaviour Survey, McKnight Risk Factor Survey; Self-report BMI.
Keery et al. (2005) [9] (USA)	Evaluated prevalence and effect of teasing by family members on body dissatisfaction, eating disturbance and psychological functioning.	372 female American adolescents. Non-clinical school population. 11- 15 years of age. *M*=12.6 *SD*=0.90.	Cross-sectional study; Regression Analysis; Appearance-related Teasing (A-RT); Self-report questionnaires: Perceptions of teasing scale – weight-teasing frequency, The Eating Disorder Inventory – BD, The Eating Disorder Inventory – DT, The Eating Disorder Inventory – B; Self-report BMI.
Olvera et al. (2013) [27] (USA)	Assessed association among parent and peer W-RT and disordered eating symptoms in a population of young adults.	141 female Hispanic and African American adolescents Healthy lifestyle / weight loss population. 9-14 years of age. *M*=11.1 *SD*=1.5 (10%<9 years)	Cross-sectional exploratory study; Regression Analysis; Weight-Related Teasing (W-RT); Self-report questionnaires: Variation of McKnight Risk Factor Survey-IV; Practitioner measured BMI.
Pearlman et al. (2019) [28] (USA)	Examined W-BT from parents and siblings in relation to disordered eating and psychosocial indices among adolescent military dependents at high risk for adult obesity and eating disorders.	128 mixed gender Military dependents. 54% female. 12-17 years of age *M*=14.35 years old, *SD*=1.55	Cross-sectional study; Exploratory analysis of covariance; Weight-Based Teasing (W-BT); Self-report questionnaires: Family Weight-Based Victimization Scale, Eating Disorder Examination interview; Self-report BMI.
Pötzsch et al. (2018) [47] (Germany)	Aimed to examine adolescents’ perceived Weight teasing and perception of adolescent and maternal perspective of weight bias	90 mixed gender adolescents OW and BED n=40, OW n=25, NW = 25 78.9% female. 12-20 years of age OW & BED – *M*=14.58 *SD*=2.39 OW – *M*=14.53 *SD*=2.55 NW – *M*= 15.84 *SD*=2.67	Cross-sectional study; Weight-Based Teasing (W-BT); self-report questionnaires: One item modified from Perception of Teasing Scale (POTS) - with parents as source of teasing; Weight Bias Internalization scale (WBIS ) and the Eating Disorder Examination-Questionnaire (EDE-Q); attitudes Towards Obese Person Scale (ATOP); Beliefs About Obese Persons Scale (BAOP); Self-report BMI.
Webb et al. (2020) [48] (Australia)	Investigated change in emotional eating while also testing the influence of social-emotional risk factors.	379 mixed gender adolescents. 56% female 0-13 years of age *M =* 12.0, *SD* = 90	Cross Sectional Prospective design; multi-level modelling, and standard multiple regression; Appearance-related Teasing (A-RT); Weight Teasing Sub-scale of Perception of Teasing Scale (POTS) - with parents and peers as source of teasing; Dutch Eating Behavior Questionnaire (DEBQ), Mood and Feelings Questionnaire, Social Anxiety Scale for Children. Practitioner measured BMI.

*A-RT* Appearance related teasing, *W-BT* weight based teasing, *W-RT*, weight related teasing, *OW* overweight, *BED* binge eating disorder, *NW* normal weight

Across the studies, eating disorders were measured using the McKnight Risk Factor Survey [26, 27], the Eating Disorder Inventory subscales [9], Youth Risk Behaviour Survey [26] and Fairburn’s 1993 Eating Disorder Examination V12 [28, 47], and Dutch Hilbert and Tuschen-Caffier 2016 variation [48]; Parental teasing was measured using the Perception of Teasing Scale [9, 47, 48], the Family-Weight Based Victimization Scale [28], and the McKnight Risk Factor Survey [26, 27]. One study additionally included specifically designed targeted teasing questions asking, “in the past year, how often has your mother made a comment about your weight or eating that made you feel bad?”, the survey included the same question for fathers and responses ranged from “never” to “always” [26].

The statistical analyses across studies offer insight to the focus of each paper and a greater understanding of the results. Three papers did not focus on prevalence, therefore reported mean and standard deviation which made it harder to compare prevalence across studies in this review. Haines et al., [26] used generalized estimating equations and jointly modelled effects of the parental and family teasing on disordered eating variables using parental teasing as a socioenvironmental predictor variable. Webb et al., [48]. used multiple regression in their primary analysis and a regression model to test whether social adversity risk factors (self-reported teasing by parents was one of the risk factors) were prospectively associated with emotional eating at T1 and T2 and presented similar patterns to Haines et al., modelling that girls receive more comments than boys overall. Pötzsch et al., [47] was the only study to include a comparator group. They used multivariate Analysis of Variance (MANOVA) to save power hypothesising group differences in stigmatizing variables including adolescents’ perceived weight teasing. Keery et al., [9] used regression analysis with parental teasing treated as a continuous variable and hierarchical regression was used to predict the power of parental teasing. Olvera et al., [27] also used hierarchical regression analysis to determine weight related teasing using parent teasing as an independent variable and elements of disordered eating as dependent variables. Pearlman et al., [28] used exploratory analysis of covariance to assess for dependent disordered eating and psychosocial functioning outcome variables.

### Prevalence of parental teasing and gender

Being able to appreciate prevalence in this field is a challenge because researchers are looking at prevalence of parental appearance or weight teasing from different constructs and via different perspectives, and therefore utilising different outcome measures resulting in the reporting of statistics that are not homogenous. Table 3 represents the within studies reports of parental teasing which ranged between 21.1 and 42%. Only two studies considered the gender of the parent [9, 27] yet without the same teasing focus. Pötzsch et al.’s, study did plan to consider the gender of the parent, but did not get sufficient fathers’ responses (*n* = 5) [47]. Keery et al., was the only study to consider appearance specific teasing and parent gender, and found that 19% of girls reported A-RT by father and 13% reported A-RT by mother [9]. Conversely, Olvera et al.’s paper was the only one to consider parent gender and W-RT [27] and found the highest reported prevalence of all the studies (42%) with 46% of weight teasing from mothers and 39% from fathers. Pearlman et al.’s 2019 W-RT study [28] did not report parent gender but overall reported 21.1% of parents teased about weight and had limited analyses about parents as they combined parent teasing with sibling teasing and focused primarily on family weight based teasing (W-BT). The largest study by Haines et al., were contacted to ask to see if prevalence results exist but did not receive a reply by the time this paper was submitted.

**Table 3:** Prevalence of reported teasing by source

Study	No in sample	Reported weight/ appearance teasing				
		Parents	Mother	Father	Siblings	Peers/classmates
Haines et al. W-RT	*N* = 10,540 Mixed Gender At risk sample	Can’t work out actual % Girls *n* = 6022 *M* = 1.3 (*SD* = 0.6) Boys *n* = 4518 *M* = 1.2 (*SD* = 0.6)	Not measured	Not measured	Not measured	Not measured
Keery et al. A-RT	*N* = 372 Girls only General Population	23%	13%	19%	29%	Not measured
Olvera et al. W-RT	*N* = 141 Girls only Minority Population	42%	46%	39%	Not measured	59%
Pearlman et al. W-BT	*N* = 128 Mixed Gender Minority Population	21.1%	Not measured	Not measured	42.5%	No measured
Potzsch et al., A-RT	*N* = 90 Mixed gender Comparison group Girls *n* = 71 78.9%	BED n = 40 *M* 7.68 (*SD* 2.38) OW n = 25 *M* 6.40 (*SD* 0.91) NW *n* = 25 *M* 6.08 (*SD* 0.40)	Not reported	Not reported	Not measured	Not measured
Webb et al., A-RT	*N* = 379 Girls *n* = 207 56% Boys n = 171 46% General population	Girls n = 207 *M* = 1.58 (*SD* = 0.89) Boys *n* = 171 *M* = 1.41 (*SD* = 0.80) Combined *M* = 1.50 (*SD* = 0.85)	Not measured	Not measured	Not measured	Girls n = 207 *M* = −0.08 (*SD* = 0.87) Boys n = 171 *M* = − 0.05 (*SD* = 0.89) Combined *M* = 1.41 (*SD* = 0.46)

Olvera et al., [27] reported more W-RT from their mothers than their fathers. Both Keery et al., [9] and Olvera et al., [27] considered the prevalence of teasing relevant to negative outcomes and found that the frequency of the teasing to be of significance. Keery et al., [9] reported higher teasing frequency was associated with poorer the outcomes. Olvera [27] also reported the girls in their study engaged with emotional eating and binge eating more as the frequency of the teasing increase.

### What parents teased about

The studies reported weight-related teasing (W-RT) [26–28] and appearance-related teasing (A-RT) [9, 47, 49]. Four of the six included studies reported on binge eating [26–28, 47]. Half the studies reported on bulimia [9, 26, 28], 33% reported on restriction [9, 28] and 50% [27, 48] reported on emotional eating. Contributing factors to eating disorders were also reported across the studies, internalization, social anxiety symptoms, depression, self-image, body dissatisfaction and weight and or shape concern.

### Influence of parental teasing

Only two papers reported a potential influence on other family members [9, 28]. Keery et al., [9] reported girls teased by family members had significantly higher levels of negative outcomes than those with no family members that tease. Parental teasing, particularly paternal teasing, was found to significantly increase the frequency of sibling teasing, which in Keery et al., [9] and Pearlman et al.’s [28] studies reported sibling teasing was 42 and 29% respectively. Olvera et al., [27] found peer teasing to be slightly more prevalent at 59% than parental teasing (42%). Keery et al., [9] found maternal teasing about weight and appearance increased risk of having a sibling who teases (*OR* = 1.37, 95% *CI* = 1.14–1.65). Yet, when paternal teasing was entered into the model, maternal teasing was no longer significant (*OR* = 1.15, 95% *CI* = .93–1.42) and paternal teasing now increased risk of sibling teasing (*OR* = 1.4, 95% *CI* = 1.16–1.70). Neither Pötzsch et al., [47], nor Webb et al., [48] considered.

### Associated eating problems

Keery et al.’s, female sample reported that both maternal and paternal A-RT was significantly associated with depression and paternal only A-RT was predictive of *eating disorders* (restriction, bulimic behaviours), and psychological outcomes (body dissatisfaction, body comparison, thin-ideal internalization and self-esteem) [9]. Similarly, Pötzsch et al. [47] found adolescents who perceived W-RT significantly predicted global eating disorder psychopathology *F* (1,82) =15.09, *p* < .001. adj *R*^*2*^ = 0.16; *b* = 0.26, *p* = <.001, 95% CI. This effect was significantly modified when considering an adolescent’s level of weight bias internalization. Webb et al.’s, most recent 2020 study found Parental W-RT was associated with emotional eating T1 (M = .22 *p* < .01) and increased over time T2 (M = .27 p < .01). In their prospective modelling one year later, self-reported appearance teasing by parents was one of two risk factors uniquely associated with emotional eating [48].

### Parent association with sibling teasing

Olvera et al., [27] conducted hierarchical regressions on W-RT and, although exploratory, found W-RT by a parent was significantly associated with eating disorders (emotional eating and binge eating). Haines et al., [26] found parental W-RT was directly associated with binge eating for both boys (OR, 1.31; 95% CI, 1.15–1.50) and girls (OR, 1.29; 95%CI, 1.08–1.55) and overweight (OR, 1.64; 95%CI, 1.36–1.96) in their cross-sectional results, yet only for girls in the prospective results. Pearlman et al., [28] found parental W-BT was significantly associated with lower self-esteem (*F* (1,115) = 8.81, *p* = 0.02), and was associated with depression although they report did not reach significance (*F* (1,116) = 3.85, *p* = 0.05).). For Pearlman, W-BT was not significantly associated with eating pathology.

## Discussion

This is the first systematic review of the association between parents as a specific source of teasing and eating disorders. Our review showed that, previous research has indicated adolescents who experienced appearance or weight teasing from parents were more likely to have eating problems and other psychopathology which has been shown to contribute to eating problems. None of the research considers this teasing to be causative but part of the equifinality of eating problems.

### Prevalence of parental teasing and gender

There are clear challenges in having discussions about appearance, weight, shape and even healthy eating without misunderstandings on what was being discussed and how it was being discussed. Parental communication is heard by the adolescent and then perceived either correctly or incorrectly as teasing, bullying or weight talk and then communicated to others in that light [11, 50]. Prevalence of appearance teasing is complicated by these concerns between intention and perception. Pötzsch et al., [47] considered maternal attitudes and although results were not the focus of this review, it is important to consider, as the Tripartite Influence Model has illustrated, the influence of the parental modelling in our prevalence results [51]. All the included studies are influenced by this perception and were self-report although none of the papers discuss family processes, studies have considered the perception of the way words are heard and have suggested there are complexities to how words are intended and how they are received, the overt and the covert communication and the potential for misunderstanding within the context of family processes [11, 12, 31, 50]. However, our findings also confirm the importance of considering the source of teasing, particularly in the family home and beyond the collective family grouping [52]. Parents are a source of appearance and/or weight related teasing and, although not the most prevalent source, their influence is clearly noteworthy. There is a paucity of research considering the gender of the parent and as the studies included in this review illustrate, there are noteworthy risk factors for mothers and daughters which would benefit from further exploration in relation to family and intrafamilial processes [9, 26, 27, 31, 47].. This is supported in broader research on parental and family comments that are not specific to teasing [13, 21, 49, 53].

### What parents teased about

Our findings specifically considered weight and appearance teasing and were consistent with other research considering parent communication not specifically teasing. Weight-related teasing was more commonly associated with males, and fathers had higher prevalence with sons than daughters [13, 54, 55]. Our findings are consistent with the wider literature on parental teasing where several studies, mostly mixed gender, have reported that A-RT increased risk factors for eating disorders, such as thin idealisation, [13, 30, 49, 50, 53–55]. In particular, Keery et al., [9] found A-RT between fathers and daughters was associated with eating disorder psychopathology.

### Influence of parental teasing

In line with previous research, our findings support the suggestion that having a parent who teased increased the risk of disordered eating directly [9, 26, 27, 47, 48] as well as indirectly through the influence of their teasing on poor social functioning, negative self-evaluation and increased appearance sensitivity and anxiety over time [9, 16, 28, 55, 56] and was considered highly influential to wellbeing outcomes [9, 26–28, 47] regardless of it not being the most reported source. A further indirect influence from parental teasing was the permission effect for siblings to view their teasing as acceptable [9, 13, 28, 53, 54]. This is important as studies have found a high prevalence of peer teasing [27] and sibling [9, 28] with negative outcomes of increased eating disorder pathology.

### Association with eating disorders

In terms of eating problems, we found binge eating, emotional eating, purging, restrictive eating through dieting and or skipping meals were associated with parental appearance or weight teasing, corroborates previous research an association with body dissatisfaction, disordered eating behaviours and unhealthy weight control behaviours [21, 36, 57–59]. However, none of the included studies sought to blame parents or to imply parents were directly responsible for disordered eating pathology.

With regards to gender and associations with eating disorders, Haines et al. [26] and Kerry et al.’s [9] findings are consistent with wider research that amongst girls that dieting [60, 61] and W-RT respectively were associated with an increased risk of disordered eating [62]. Amongst boys there were fewer risk factors that had a shared effect on weight related problems which is consistent with Neumark-Sztainer et al.’s findings [63].

In contrast Olvera et al., [27] found W-RT is related to be associated with only weight concern and eating problems (consistent with wider research [63] but not with dieting and skipping meals [9, 21, 28, 64]. Possible explanations for this difference is the specific weight focused cohort and cultural differences between the samples.

Taken together, the findings reported here suggest parental conversations are important and may impact adolescent self-schema and therefore behaviour and coping strategies. None of the papers however examined parental intention or family processes and no causal conclusions can be drawn. Our findings are consistent with three meta-analyses conducted in this broad field of research [40, 42, 65]. One study by Menzel et al., [40] considered appearance-related teasing, body dissatisfaction, and disordered eating and support inclusion of intervention programs that focus on handling negative, appearance-related commentary. A second meta-analysis by Gillison et al., [65] did not focus on teasing but on weight commentary and equally supported the findings that encouraging positive parental communication around weight conversations is important. The third, a systematic review and meta-analysis specifically looked at bullying and teasing and associations with eating disorders and highlighted the importance of considering the source of the teasing. All authors call for more longitudinal research.

### Limitations and strengths

A notable limitation of research identified in this review is that all studies included are cross sectional which limits the ability to investigate causal pathways. Every study (*N* = 6) used self-report questionnaires, which is both, a strength in terms of a resource efficient way to collect large amounts of measurable data, but a limitation due to the potential for reporting bias. All papers considered adolescent reports only which could be influenced by recall bias as well as by the quality of relationship with their parent which could influence how the words were received and none of the included studies considered the influence of family processes. Similarly, the self-reporting of BMI across most studies may also be considered fraught with reporting biases that might influence findings, however this is a common criticism of much general population research in eating problem studies and the findings are not substantially different in studies that used a practitioner to collect the BMI data. The lack of gender balance and the possible influence resulting from the inclusion of one large cohort study means future research should include gender comparisons. The lack of mixed-method and qualitative papers meant careful and in-depth investigations of the language, or discourse, itself was not well understood.

In addition to the limitations identified amongst the methodologies utilised by the studies captured in this review it is also pertinent to consider the limitations of the review process itself. For instance, the review focused very specifically on the word ‘teasing’ in relation to parents and this may have limited our ability to consider studies with other forms of communication such as healthful eating or this misunderstanding of parental humour and communication that could be taken as critical or influential by an adolescent. Although every effort was made to capture all literature in this field by hand searching relevant articles, our highly specific search phrase may have lost some sensitivity.

Strengths of the review include being able to consider French language papers; including grey literature and theses; contacting authors of papers that had an unclear number of participants outside our inclusion criteria for age; having multiple readers and agreeing on the quality and inclusion of 100% of the included papers.

In summary, the studies included in this review focused on parental appearance and weight-related teasing, and associations to eating problems in adolescents. This review found significant associations between parent appearance and weight teasing and eating disorders (binge eating, bulimia, emotional eating, weight control behaviours, restriction) and psychosocial contributors to eating disorders (self-esteem, depression, perfectionism, self-image, body dissatisfaction, weight concern, internalization and comparison). Notably, this was regardless of the intention by the parent and once again, emphasis must go on the purpose of this review to collate the existing data to see what further research is needed to better understand the influence of communication within the family processes. It is essential to establish the frequency and impact of inadvertent parental weight shape and or body teasing and what the consequences of this teasing could be on adolescents so support can be offered to parents on how to have healthful conversations around weight shape and eating. This synthesis of the findings offers parents more awareness around the words and humour used either directed at their children or around their children. The implications include consideration of the protective nature of healthful eating conversations and the risk of humour in parent and family health programs and in other public health measures to reduce the risk of eating and weight disorders. This is because, without adequate skills to understand the nuances of communication and humour from parents, adolescents may be more likely to find problem coping strategies which such as emotional eating or food restriction, and emotional avoidance strategies; the consequence of those problem coping strategies include appearance-rejection sensitivity, body dysmorphic disorder, disordered eating, emotional eating, binge eating, anorexia, and depression [17–19]. Furthermore, we know that healthy weight talk and supportive, positive parent-adolescent relationships have been considered protective for eating disorder pathology and protective against the potential negative impact of peer influences [66].

## Conclusion

Every study included in this review considered parental teasing to be a concern, not only for the direct impacts but also the indirect as it may be associated with increased emotional health problems. Therefore, apportioning blame on parents is not the intention of this review and should not be for future studies. This review found that in research specific to parental appearance teasing there is a likely association with adolescent ‘eating problems’ however, there are limitations to the research to date. The impact of parental teasing is likely inadvertent and thereby responsive to change, therefore future research should employ representative longitudinal designs to develop a greater understanding of the direction of relationships between verbal and non-verbal communications, how and why they are perceived and dealt with by adolescents and consider the gendered source of the parental teasing within at family processes context. This review also echoes the call for a reduction in the acceptability of appearance and weight related teasing within family, the community and society at large.

## Supplementary Information

**Additional file 1.** Search History by Database. A record of the search terms for each database

**Additional file 2.** Variation Downs and Black Checklist. This is the variation of the Downs and Black checklist used for analysis of included.

**Additional file 3.** Reference. Reference list of included articles

## Data Availability

All data included in this systematic review is from previously published papers. No new datasets were produced that have not been included in this journal submission.

## Abbreviations

WHO: World Health Organisation
A-RT: Appearance Related Teasing
W-RT: Weight Related Teasing
W-BT: Weight Based Teasing
ED: Eating Disorder
BE: Binge Eating
BMI: Body Mass Index
BDQI: Downs and Black Quality Index
