# Supplementary material for: Parental appearance teasing in adolescence and associations with eating problems: a systematic review
Source: BMC Public Health. 2021 Mar 6;21:450. doi: 10.1186/s12889-021-10416-5 (PMC7936414; doi:10.1186/s12889-021-10416-5)
Supplement: Supplementary file 1 — Additional file 1. Search History by Database. A record of the search terms for each database [file 12889_2021_10416_MOESM1_ESM.docx]

| Database | Search terms used | Language Inclusion | Results | Duplicates | Reason for excluding by title or abstract | Relevant Abstracts to consider |
| --- | --- | --- | --- | --- | --- | --- |
| Scopus | (( TITLE-ABS-KEY ( adolescen* )  OR  TITLE-ABS-KEY ( teen* )  OR  TITLE-ABS KEY ( youth* ) )  AND  PUBYEAR  >  1979 )  AND  ( ( TITLE-ABS KEY ( teasing )  AND  TITLE-ABS KEY ( appearance  OR  body  OR  shape ) ) AND  PUBYEAR  >  1979 )  AND  ( ( TITLE-ABS-KEY ( parent* )  OR  TITLE-ABS-KEY ( maternal )  OR  TITLE-ABS- KEY ( paternal ) )  AND  PUBYEAR  >  1979 )  AND  ( ( TITLE-ABS-KEY ( "eating problems" )  OR  TITLE-ABS-KEY ( anorexia )  OR  TITLE-ABS-KEY ( bulimia )  OR  TITLE-ABS-KEY ( "eating problems" ) )  AND  PUBYEAR  >  1979 ) | English  French | 13 | 0 | 5 x not specifically parent/teasing  5 x not relevant cohort | 3 |
| EBSCOhost  PsycInfo | aedolescen*, teen* youth*; parent*, maternal, paternal, mother*, father*; teas*, bull*, DE Teasing, DE bullying, DE jokes;DE Physical appearance, DE Physical attractiveness. DE Physique, DE Body weight, DE Body size, appearance, weight, shape; DE eating disorders, DE anorexia nervosa, DE binge eating disorder, DE bulimia, DE purging (eating disorders), (eat* N3(problem* or disorder* or binge* or behavio#r*) | English  French | 45 | 17 | 26 x not specifically parent/teasing  1 x not relevant cohort  1 x not disordered eating | 0 |
| EBSCOhost  CINAHL | MH Adolescent behaviour, Adolescent psychology, adolescen*, teen* youth*; MH Parent-child relations, MH parent behaviour: parent, maternal, paternal, mother, father; MH bullying, MH verbal abuse, teas*, bull*; MH personal appearance, body, appearance, weight, shape; MH eating disorders, MH binge eating disorder, MH Bulimia Nervosa, (eat* N3(problem* or disorder* or binge* or behavio#r*) anorexia, bulimia | English  French | 14 | 12 | 2 x not relevant cohort | 0 |
| Ovid Medline | Parent*, maternal, paternal, mother, father; exp parents, exp fathers, exp mothers; weight teasing, shape teasing, body teasing, teas*, bull*, appearance teasing, body image, body dissatisfaction, physical appearance, exp. bullying; exp feeding and eating disorders, anorexia nervosa, avoidant restrictive food intake disorder, binge eating disorder, bulimia nervosa, binge eating, eating problems, eating disorder; adolescen*, teen* youth*; exp. Adolescent Behavior or Adolescent | English  French | 26 | 7 | 14 x not specifically parent/teasing  3 x not relevant cohort | 2 |
| Google Scholar | [parent appearance weight teasing adolescent eating disorders](https://scholar.google.com/scholar?q=parent+appearance+weight+teasing+adolescent+eating+disorders&hl=en&as_sdt=0,5&scilib=1025&scioq=parent+appearance+weight+teasing+adolescent+eating+disorders)  Considered first 4 pages (20 results per page) | English  French | 80 | 10 | 65 x not specifically parent/teasing  2 x not relevant cohort  2 x not disordered eating | 1 |
