# Supplementary material for: Parental appearance teasing in adolescence and associations with eating problems: a systematic review
Source: BMC Public Health. 2021 Mar 6;21:450. doi: 10.1186/s12889-021-10416-5 (PMC7936414; doi:10.1186/s12889-021-10416-5)
Supplement: Supplementary file 2 — Additional file 2. Variation Downs and Black Checklist. This is the variation of the Downs and Black checklist used for analysis of included. [file 12889_2021_10416_MOESM2_ESM.docx]

**Variation Downs and Black (1998) checklist for the assessment of methodological quality**

| **Aspects** | **Check list** | **Almenara & Jezek 2015** | **Haines, et al. 2010** | **Keery et al., 2005** | **Olvera et al., 2013** | **Pearlman et al. 2019** | **Potzsch et al., 2018** | **Webb et al., 2020** |
| --- | --- | --- | --- | --- | --- | --- | --- | --- |
| **Reporting** | Are the hypothesis/aim/objectives of the study clearly described? | 1 | 1 | 1 | 1 | 1 | 1 | 1 |
|  | Are the main outcomes to be measured clearly described in the Introduction or Methods section? | 1 | 1 | 1 | 1 | 1 | 1 | 1 |
|  | Are the characteristics of the participants included in the study clearly described? | 1 | 1 | 1 | 1 | 1 | 1 | 1 |
|  | Is the exposure weight shape or eating teasing? | 1 | 1 | 1 | 1 | 1 | 1 | 1 |
|  | Are the parents reported to be responsible for the weight, shape or eating related teasing? | 1 | 1 | 1 | 1 | 1 | 1 | 1 |
|  | Are the adolescents participating in the study between 10 and 19 years of age? | 1 | 1 | 1 | 0 | 1 | 0 | 0 |
|  | Are the analyses and controls for confounds appropriate? | 1 | 1 | 1 | 1 | 1 | 1 | 1 |
|  | Are the main findings of the study clearly described? | 1 | 1 | 1 | 1 | 1 | 1 | 1 |
|  | Does the study provide estimates of the random variability in the data for the main outcomes? | 1 | 1 | 1 | 1 | 1 | 1 | 1 |
| **External validity** | Were the subjects asked to participate in the study representative of the entire population from which they were recruited, i.e. were response rates acceptable (at least 50%)? | 1 | 0 | 0 | 0 | 0 | 0 | 0 |
|  | Were the statistical tests used to assess the main  outcomes appropriate? | 1 | 1 | 1 | 1 | 1 | 1 | 1 |
|  | Were the main outcome measures used accurate (valid and reliable)? | 1 | 1 | 1 | 1 | 1 | 1 | 1 |
| **Internal Validity** | Was there adequate adjustment for confounding in the analyses from which the main findings were drawn? | 1 | 1 | 1 | 1 | 1 | 1 | 1 |
| **Power** | Did the study have sufficient power to detect a clinically important effect where the probability value for a difference being due to chance is less than 5%, and/or power analyses reported? | 1 | 1 | 1 | 1 | 1 | 1 | 1 |
| **Other** | Was it an original Study? | 1 | 1 | 1 | 1 | 1 | 1 | 1 |
|  | Did the study use a standardized measure of disordered eating | 0 | 1 | 1 | 1 | 1 | 1 | 1 |
|  | Score | 15 | 15 | 15 | 14 | 15 | 14 | 14 |

Yes = 1 No = 0 Unable to determine = 0
