# Supplementary material for: Parental appearance teasing in adolescence and associations with eating problems: a systematic review
Source: BMC Public Health. 2021 Mar 6;21:450. doi: 10.1186/s12889-021-10416-5 (PMC7936414; doi:10.1186/s12889-021-10416-5)
Supplement: Supplementary file 3 — Additional file 3. Reference. Reference list of included articles [file 12889_2021_10416_MOESM3_ESM.docx]

Included Studies

Keery H, Boutelle K, van den Berg P, Thompson JK. The impact of appearance-related teasing by family members. Journal of Adolescent Health. 2005;37(2):120-7.

Haines J, Hannan P, van Den Berg P, Eisenberg M, Neumark-Sztainer D. Weight-Related Teasing from Adolescence to Young Adulthood: Longitudinal and Secular Trends between 1999 and 2010. Obesity. 2013;21(9):E428-E34.

Olvera N, Dempsey A, Gonzalez E, Abrahamson C. Weight-related teasing, emotional eating, and weight control behaviors in Hispanic and African American girls. Eating Behaviors. 2013;14(4):513-7.

Pearlman AT, Schvey NA, Neyland MKH, Solomon S, Hennigan K, Schindler R, et al. Associations between Family Weight-Based Teasing, Eating Pathology, and Psychosocial Functioning among Adolescent Military Dependents. International Journal of Environmental Research and Public Health. 2019;17(24).

Pötzsch A, Rudolph A, Schmidt R, Hilbert A. Two sides of weight bias in adolescent binge‐eating disorder: Adolescents’ perceptions and maternal attitudes. International Journal of Eating Disorders. 2018;51(12):1339-45.

Webb HJ, Kerin JL, Zimmer-Gembeck MJ. Increases in Emotional Eating During Early Adolescence and Associations With Appearance Teasing by Parents and Peers, Rejection, Victimization, Depression, and Social Anxiety. The Journal of early adolescence. 2020:27243162095046.
